# Sphingosine Kinase 1 and Sphingosine 1-Phosphate Receptor 3 Are Functionally Upregulated on Astrocytes under Pro-Inflammatory Conditions

**DOI:** 10.1371/journal.pone.0023905

**Published:** 2011-08-24

**Authors:** Iris Fischer, Chantal Alliod, Nicolas Martinier, Jia Newcombe, Corinne Brana, Sandrine Pouly

**Affiliations:** 1 TA Neurodegenerative Diseases, Geneva Research Center, Merck Serono International, Geneva, Switzerland; 2 NeuroResource, UCL Institute of Neurology, London, England; University of North Dakota, United States of America

## Abstract

**Background:**

Reactive astrocytes are implicated in the development and maintenance of neuroinflammation in the demyelinating disease multiple sclerosis (MS). The sphingosine kinase 1 (SphK1)/sphingosine1-phosphate (S1P) receptor signaling pathway is involved in modulation of the inflammatory response in many cell types, but the role of S1P receptor subtype 3 (S1P_3_) signaling and SphK1 in activated rat astrocytes has not been defined.

**Methodology/Principal Findings:**

Using immunohistochemistry we observed the upregulation of S1P_3_ and SphK1 expression on reactive astrocytes and SphK1 on macrophages in MS lesions. Increased mRNA and protein expression of S1P_3_ and SphK1, as measured by qPCR and Western blotting respectively, was observed after treatment of rat primary astrocyte cultures with the pro-inflammatory stimulus lipopolysaccharide (LPS). Activation of SphK by LPS stimulation was confirmed by SphK activity assay and was blocked by the use of the SphK inhibitor SKI (2-(p-hydroxyanilino)-4-(p-chlorphenyl) thiazole. Treatment of astrocytes with a selective S1P_3_ agonist led to increased phosphorylation of extracellular signal-regulated kinase (ERK)-1/2), which was further elevated with a LPS pre-challenge, suggesting that S1P_3_ upregulation can lead to increased functionality. Moreover, astrocyte migration in a scratch assay was induced by S1P and LPS and this LPS-induced migration was sensitive to inhibition of SphK1, and independent of cell proliferation. In addition, S1P induced secretion of the potentially neuroprotective chemokine CXCL1, which was increased when astrocytes were pre-challenged with LPS. A more prominent role of S1P_3_ signaling compared to S1P_1_ signaling was demonstrated by the use of selective S1P_3_ or S1P_1_ agonists.

**Conclusion/Significance:**

In summary, our data demonstrate that the SphK1/S1P_3_ signaling axis is upregulated when astrocytes are activated by LPS. This signaling pathway appears to play a role in the establishment and maintenance of astrocyte activation. Upregulation of the pathway in MS may be detrimental, e.g. through enhancing astrogliosis, or beneficial through increased remyelination via CXCL1.

## Introduction

Astrocytes are the most abundant glial cells in the mammalian central nervous system (CNS). They have important functions in maintenance of homeostasis and are involved in synaptic function and physical structuring of the CNS during development [1], and become reactive in response to pathological insults [2], [3]. They can upregulate genes involved in amplification of inflammation by attracting inflammatory cells to specific sites and limit immune cell invasion of adjacent healthy parenchyma [4], [5]. In inflammatory demyelinating multiple sclerosis (MS) lesions, reactive astrocytes present a hypertrophic phenotype and form astroglial scars.

Astrocytes can be activated *in vitro* by various stimuli such as LPS, a bacterial polysaccharide commonly used as a pro-inflammatory stimulus which signals mainly through the Toll-like receptor (TLR) 4. LPS activates the sphingosine kinase 1/sphingosine-1-phosphate (SphK1/S1P) signaling axis in other cell types including microglia [6] and macrophages [7], leading to translocation of SphK1 to the plasma membrane where it converts its substrate sphingosine to the bioactive sphingolipid S1P [8]–[10]. S1P can elicit a wide variety of cellular responses including inflammation and can act intracellularly as a second messenger or extracellularly by binding to the G protein-coupled receptors S1P1 to S1P5 [11], [12]. In the CNS, S1P is involved in induction of astrocyte proliferation, migration and survival [13], and was found to be increased in the cerebrospinal fluid of MS patients, suggesting its involvement in chronic neuroinflammation [14]. SphK1 CNS expression is increased by kainic acid or hypoxia [15], [16], and probably results in increased production of S1P. However, the expression of SphK1 in MS CNS tissues has not been reported.

All S1P receptors except S1P_4_ are expressed in the CNS as assessed at the mRNA level. In experimental autoimmune encephalomyelitis (EAE), an animal model commonly used to study inflammatory aspects of MS, S1P receptors were shown to be differentially regulated as S1P_1_ and S1P_5_ mRNAs were downregulated at days 11 and 29 in spinal cord, whereas S1P_4_ and S1P_3_ mRNAs were upregulated [17]. The increase of S1P_4_ expression probably resulted from infiltration of immune cells. The S1P_3_ receptor is expressed by several CNS cell types including astrocytes, microglia and neurons, and by immune cells such as dendritic cells and B lymphocytes [18], [19]. The increased expression of this receptor during EAE could therefore be due either to immune cell infiltration or upregulation by CNS cells, or both. In normal CNS tissues, astrocytes were reported to express mainly S1P_3_ and S1P_1_ mRNA, with very low levels of S1P_2_ and S1P_5_ [20]–[22].

FTY720 is a S1P analogue that has recently been approved as an anti-inflammatory therapy for MS. It is a pro-drug that needs to be phosphorylated to FTY720P by sphingosine kinase 2 (SphK2) to become active and act as agonist of S1P1, 3, 4 and 5 receptors [23], [24]. It decreases immune cell trafficking by activating S1P_1_ on lymphocytes, thereby inducing internalization and degradation of these receptors [25]–[27], which in consequence leads to sequestration of the lymphocytes in lymph nodes by preventing them from following the S1P gradient that would guide them into the bloodstream [28]. The resulting lymphopenia decreases the migration of immune cells into the CNS. FTY720 readily crosses the blood-brain barrier [29], and it may exert additional effects in the CNS which would probably be mediated via S1P_1_, S1P_3_ and S1P_5_, although there is no strong evidence demonstrating direct neuroprotective effects.

Until recently the expression of S1P_3_ on astrocytes *in vitro* or in neuroinflammatory disorders and the roles of this signaling axis in CNS inflammation had not been investigated, but van Doorn and colleagues [30] have demonstrated S1P_3_ upregulation in MS tissues.

In the present study, we have confirmed this observation and shown for the first time the enhanced expression of SphK1 on macrophages and astrocytes in MS lesions. We have further showed increased expression and functionality of both SphK1 and S1P_3_ in cultures of activated rat astrocytes, which demonstrated the potential therapeutic impact of targeting this signaling axis in MS.

## Results

### S1P_3_ and SphK1 are expressed on reactive astrocytes in MS lesions

**Figure 1:**
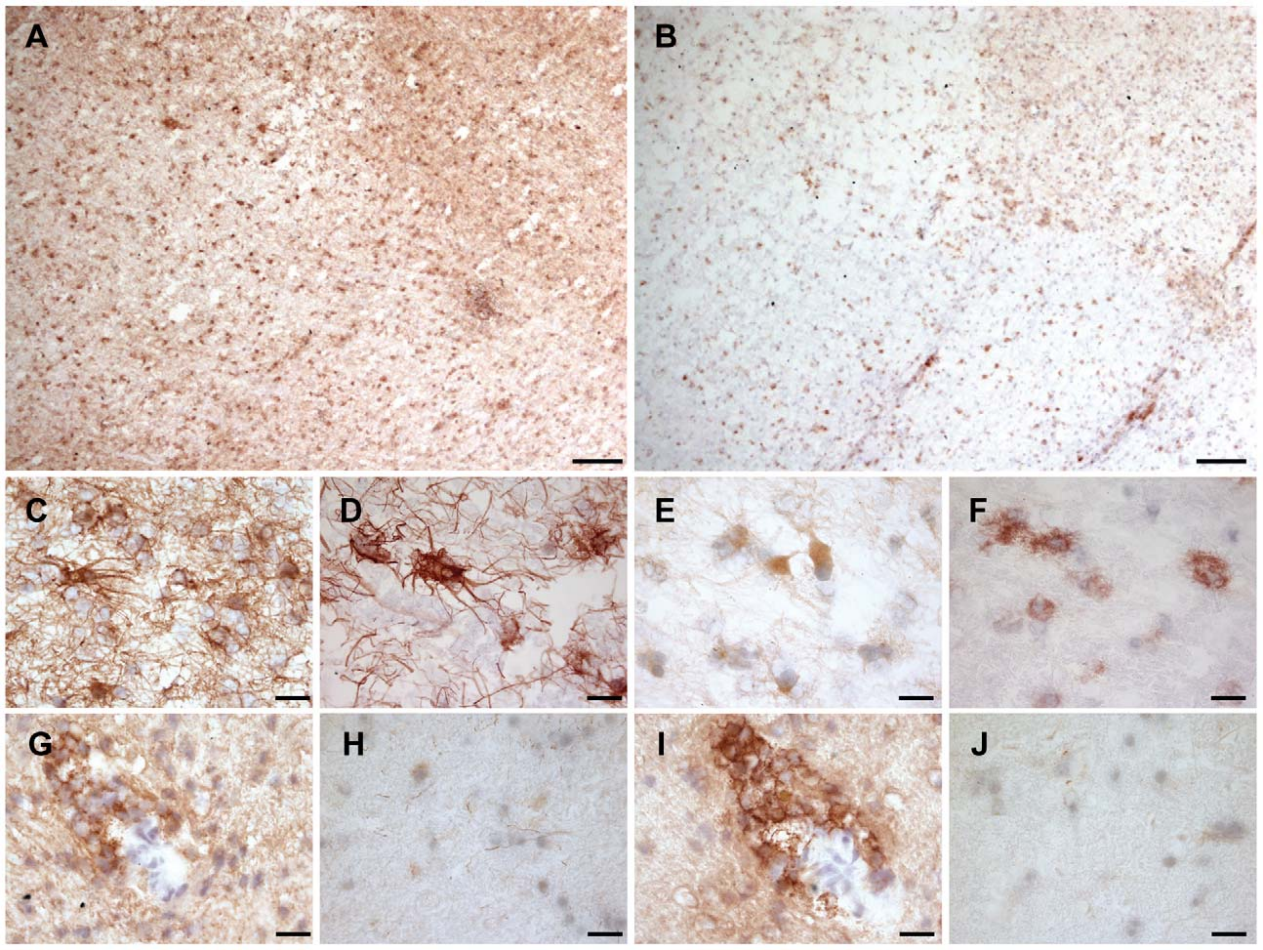
S1P_3_ and SphK1 are upregulated in MS lesions. (A, B): Immunohistochemical peroxidise staining shows that S1P_3_ receptor and SphK1 enzyme expressions are increased in a chronic-active MS lesion which was located in parietal subventricular white matter. S1P_3_ expression is strong on reactive astrocytes in this lesion border (C), in the lesion (D) and on perivascular cells (G), but is weak in normal control brain white matter (H). SphK1 expression is increased in reactive astrocytes (E) and in macrophages in this MS lesion (F). A particularly high expression of SphK1 is seen in perivascular inflammatory cells (I), whereas its expression is very low in normal control brain white matter (J). This Figure shows representative stainings. Scale bars are 200 µm for A and B, and 20 µm for C–J. The sections were counterstained with haematoxylin.

**Figure 2:**
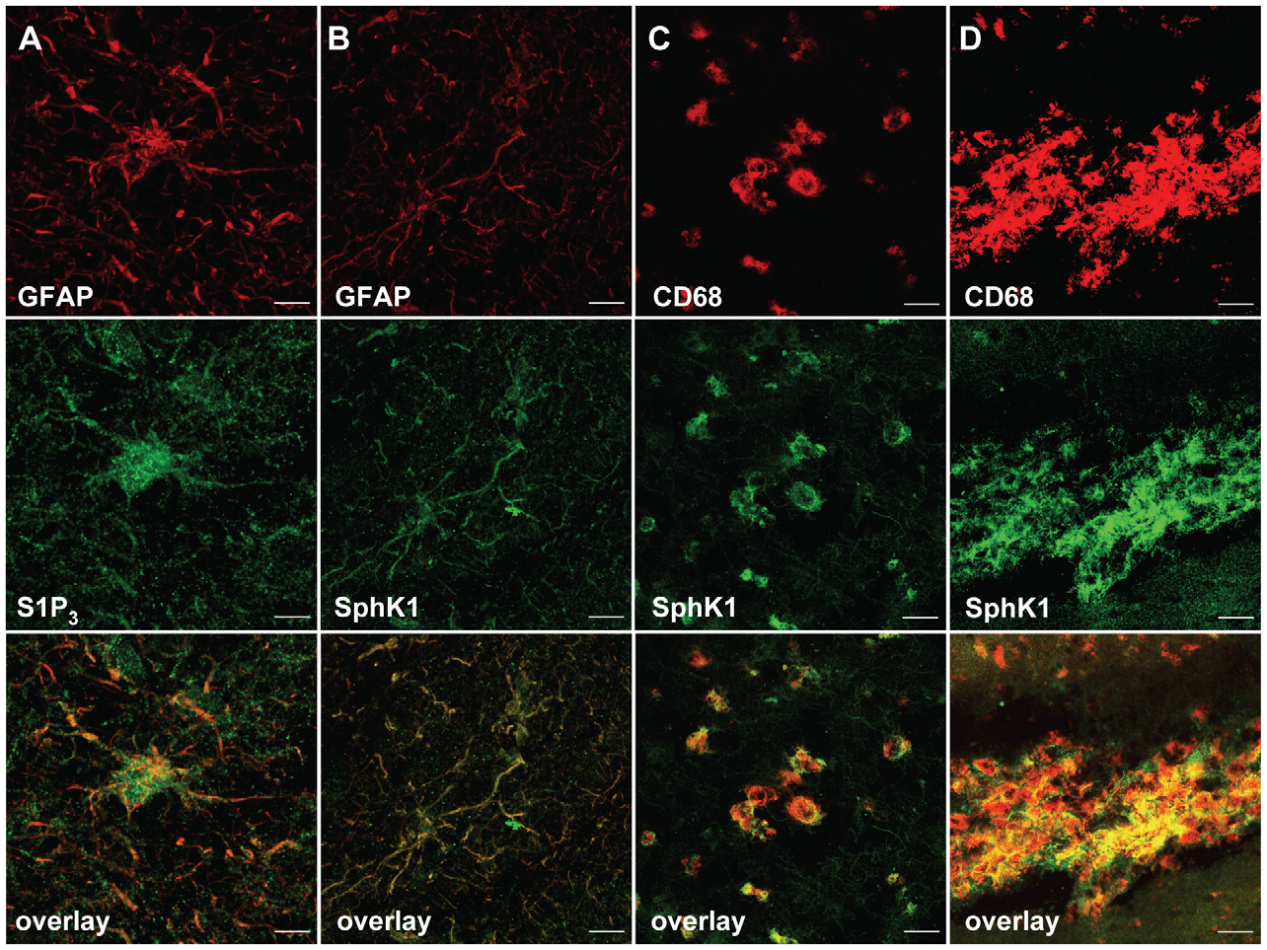
S1P_3_ and SphK1 are expressed by reactive astrocytes and macrophages in MS lesions. Immunofluorescence co-localization studies confirmed that S1P_3_ is predominately expressed by reactive astrocytes (A). SphK1 is also expressed on reactive astrocytes (B), but the major cell type expressing SphK1 is macrophage in lesions and perivascular cuffs (C–D). Scale bars are 10 µm.

Eight MS lesions from seven patients, and scored as actively demyelinating, chronic-active or chronic-inactive were analyzed by immunohistochemistry. Expression of the receptor S1P_3_ was especially strong on reactive astrocytes in plaques and their lesion borders (Fig. 1A, C, D). Expression of S1P_3_ was also detected in perivascular inflammatory cells in cuffs within plaques and also in surrounding tissue (Fig. 1G). In comparison, only weak expression of S1P_3_ was detected in normal control brain (Fig. 1H). Co-expression of S1P_3_ and glial fibrillary acidic protein (GFAP) on all reactive astrocytes in MS lesions and surrounding tissues was confirmed by double-immunostaining (Fig. 2A), but only small numbers of S1P_3_-expressing CD68-positive macrophages could also be detected (data not shown).

We also found an increased expression of SphK1 in MS lesions (Fig. 1B), with only very weak expression of the enzyme in normal control brain (Fig. 1J). This increase was particularly marked on cells with a macrophage-like morphology in plaques and their borders (Fig. 1F). Expression of SphK1 was also increased on reactive astrocytes (Fig. 1E) in the same areas, and in perivascular inflammatory cell cuffs (Fig. 1I). Co-expression of SphK1 and GFAP on reactive astrocytes in MS lesions and their borders was confirmed by double immunostaining (Fig. 2B), and SphK1 expression on macrophages in MS lesions, lesion borders and around blood vessels was confirmed by double immunostaining for SphK1 and CD68 (Fig. 2C, D).

### S1P_3_ and SphK1 mRNA and protein are increased in activated astrocytes in vitro

**Figure 3:**
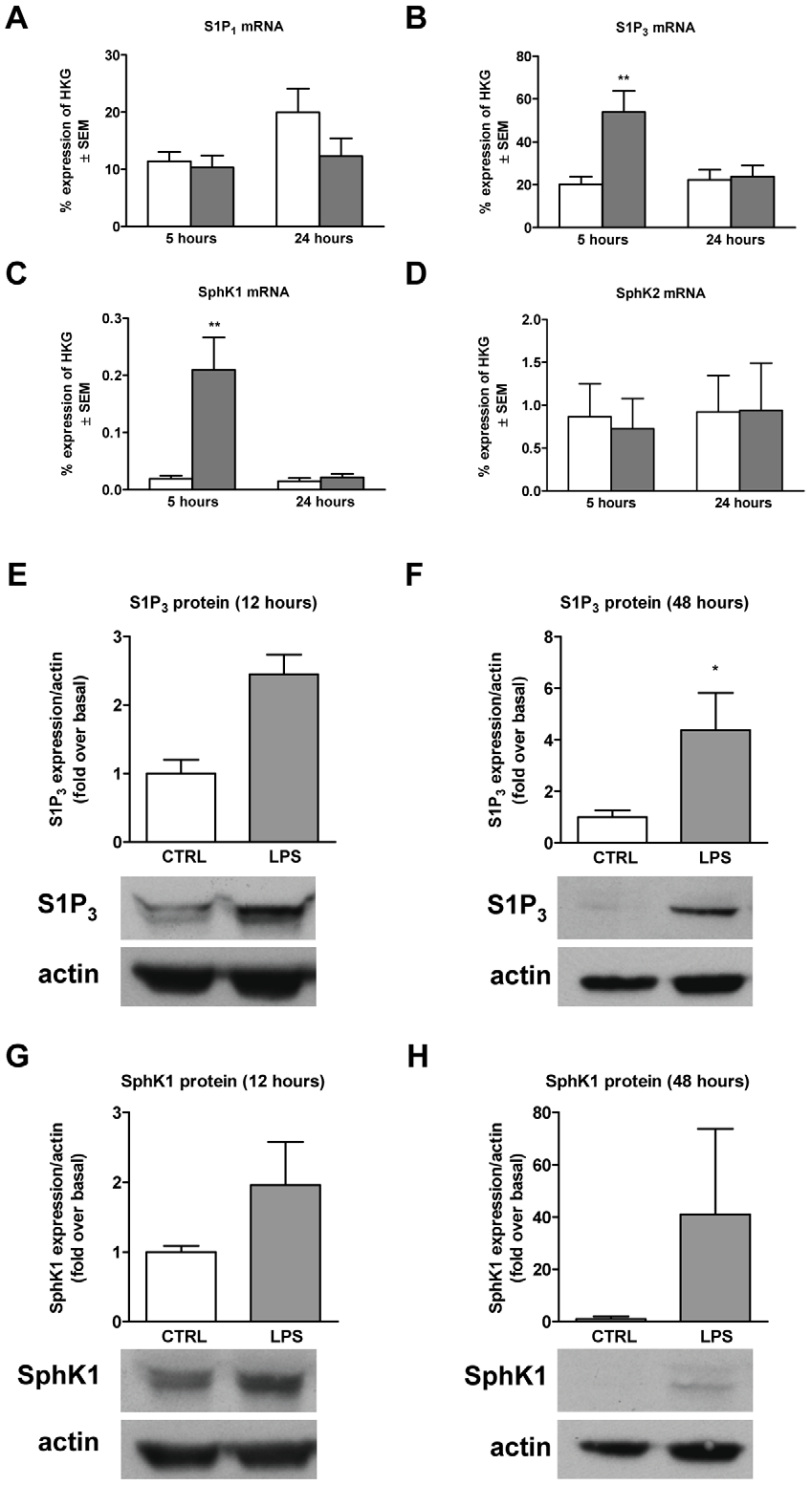
S1P_3_ and SphK1 mRNAs and protein are upregulated in rat primary astrocytes by LPS stimulation. Primary astrocytes were incubated in culture medium for 5 or 24 h with LPS (100 ng/ml). The mRNA levels of S1P_1_ (A), S1P_3_ (B), SphK1 (C) and SphK2 (D) were assessed after 5 h and 24 h incubation. Quantitative PCR results are shown as the percentage expression of HKG (GAPDH) and represent mean ± SEM of three independent experiments. LPS mediated sustained upregulation of S1P_3_ (E–F) and SphK1 (G–H) as shown by Western blots of plasma membrane fractions. Primary rat astrocytes were incubated with LPS (100 ng/ml) in serum-free medium containing 0.25% BSA for 12 and 48 h. Representative immunoblots are shown. Graphs represent the mean ± SEM of three independent experiments and are reported as protein expression normalized to actin, expressed as fold change over basal level. One-Way ANOVA followed by Bonferroni's multiple comparison test: *p<0.05, **p<0.01 vs. respective control.

To test whether the upregulation of S1P_3_ receptor and the enzyme SphK1 observed in MS lesions can be induced *in vitro* under pro-inflammatory conditions, primary rat astrocytes were activated with the pro-inflammatory stimulus LPS (100 ng/ml) for 5 h and 24 h. This concentration of LPS was found to induce maximal response of the astrocytes (data not shown). The mRNA level of S1P_3_ was significantly upregulated after a 5 h incubation with LPS (3-fold) compared to untreated cells (Fig. 3B), and SphK1 was upregulated by 10-fold (Fig. 3C). At 24 h post-stimulation, expression of S1P_3_ and SphK1 was back to basal level. Neither S1P_1_ nor SphK2 were regulated by the LPS (Fig. 3A, D). The increase of S1P_3_ and SphK1 was confirmed at the protein level in membrane preparations following LPS challenge. Both S1P_3_ receptor and SphK1 were upregulated on astrocyte plasma membranes in response to LPS challenge after a 12 h treatment (Fig. 3E, G), which was maintained for at least 48 h (Fig. 3F, H).

### LPS induces SphK1 activity and increases S1P_3_ receptor signaling

**Figure 4:**
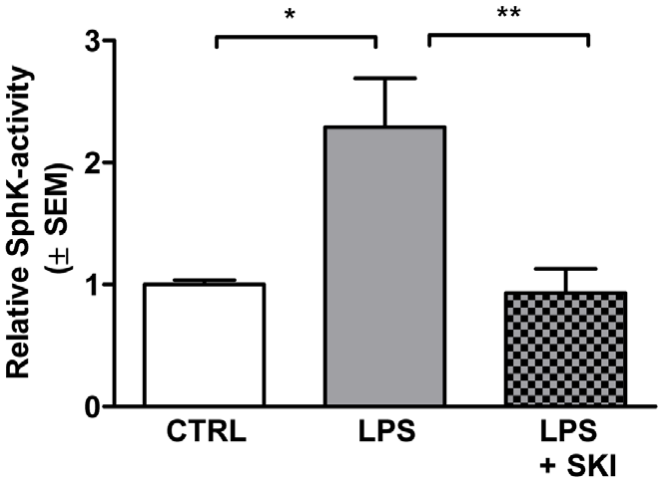
SphK1 is activated in response to LPS. Serum-deprived astrocytes were incubated for 30 min with 100 ng/ml LPS in the presence or absence of SKI (10 µg/ml). 100 µg of cell extracts were used to determine SphK1 activity by thin layer chromatography. Data represents the mean ± SEM of three independent experiments. One-Way ANOVA followed by Bonferroni's multiple comparison test: *p<0.05, **p<0.01.

The upregulation of SphK1 on plasma membranes indicates that the enzyme was translocated and thus activated in response to stimulation with LPS. This was confirmed by thin layer chromatography, using extracts of serum-deprived cells stimulated or not with LPS (100 ng/ml) for 30 min. SphK1 activity was significantly increased in LPS-stimulated astrocytes, and pre-incubation with SKI (10 µg/ml), described as a selective SphK1 inhibitor [31], reversed the activity of the enzyme to the basal level (Fig. 4A).

**Figure 5:**
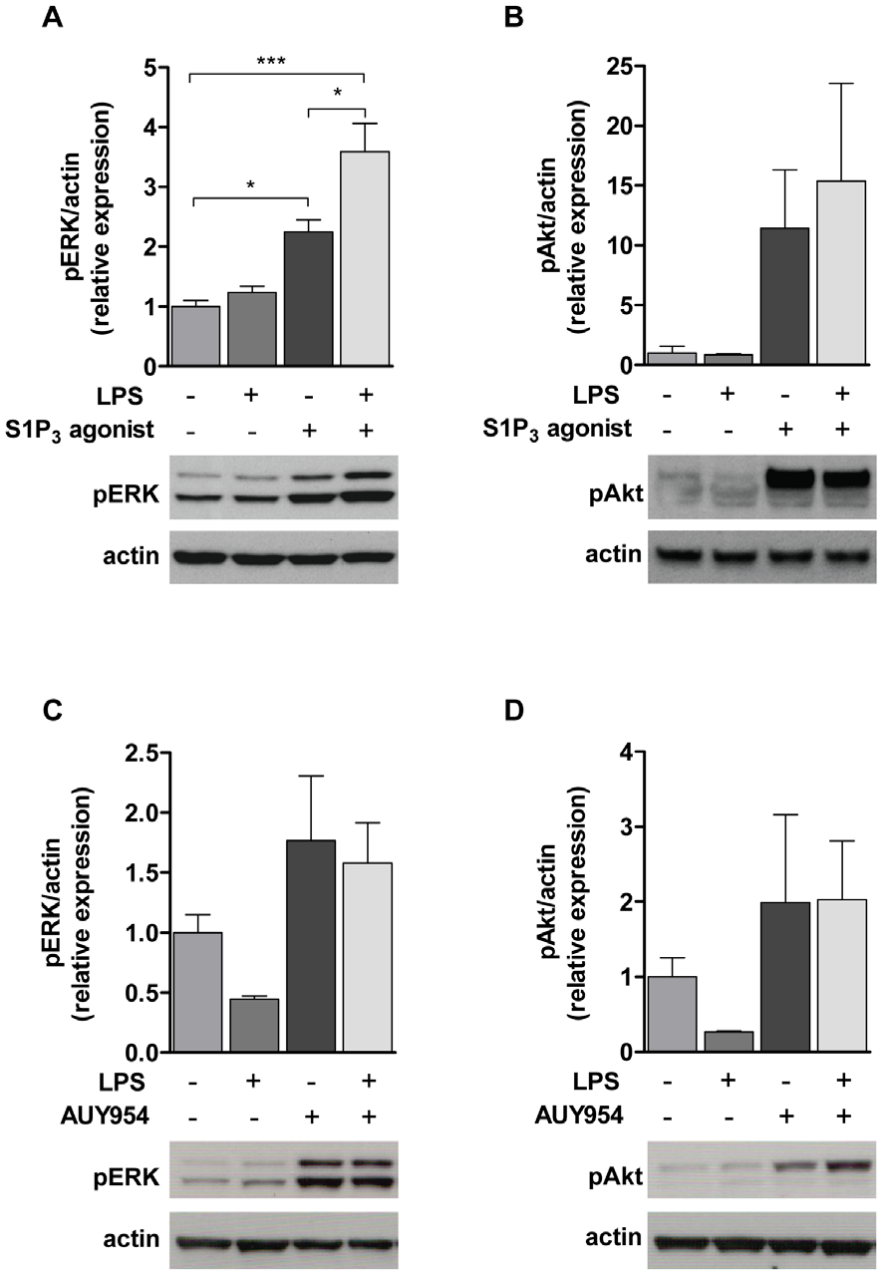
Increased S1P_3_-mediated ERK-1/2, but not Akt signaling by LPS. Astrocytes were serum-deprived during the 12 h treatment with LPS and were then stimulated for 20 min with 10 µM S1P_3_ agonist (Compound 20) or 1 µM AUY954. The results show the relative ERK-1/2 phosphorylation (A, C ) and Akt phosphorylation (B, D) ± SEM normalized against actin for three (A, B) and two (C, D) independent experiments. One-Way ANOVA followed by Bonferroni's multiple comparison test: *p<0.05, **p<0.01, ***p<0.001.

Phosphorylation of ERK-1/2 and Akt, two signaling pathways downstream of S1P receptors, was then assessed following stimulation with the S1P_3_ specific agonist Compound 20, a dicyclohexylamide molecule which showed an agonistic activity on S1P_3_ (EC50 = 350 nM), while not activating any other S1P receptor (EC50s>40 µM) [32]. Primary rat astrocytes were pre-incubated with 100 ng/ml LPS for 12 h in serum-free medium and then stimulated with the S1P_3_ agonist (Compound 20, 10 µM) for 20 min. LPS treatment alone for 12 h did not affect ERK-1/2 phosphorylation compared to untreated controls (Fig. 5A). Stimulation with the S1P_3_ agonist (Compound 20), increased ERK-1/2 phosphorylation by two-fold in cells not treated with LPS and was further elevated in cells pre-challenged with LPS. Phosphorylation of Akt was also induced by treatment with the S1P_3_ agonist, but statistical significance was not reached (Fig. 5B). However, in the case of pre-activation with LPS, very little or no elevation of Akt phosphorylation was observed with the S1P_3_ agonist when compared to the non-activated cells.

In contrast, when using the specific S1P_1_ receptor agonist AUY954, no further increase in ERK-1/2- or Akt-phosphorylation levels was observed in LPS-stimulated cells compared to the phosphorylation mediated by the agonist alone (Fig. 5C, D).

### Involvement of SphK1 activity in LPS-induced astrocyte migration

**Figure 6:**
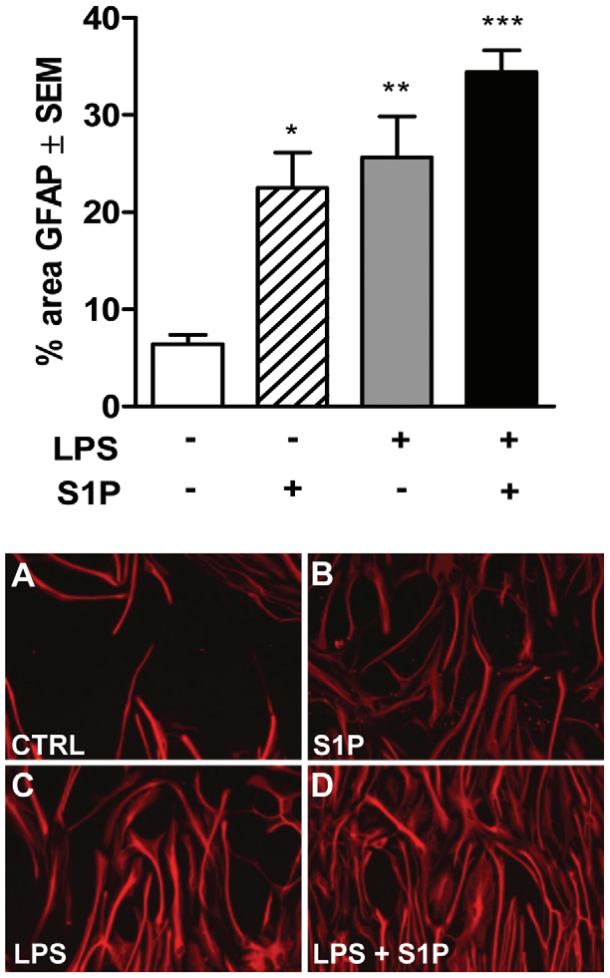
S1P and LPS induce astrocyte migration in a scratch assay. Astrocytes were stimulated with S1P (500 nM), LPS (100 ng/ml) or combined treatments of S1P+LPS. Cells were allowed to migrate into the scratch for 48 h. The images show the representative migration of astrocytes into a scratch in response to the different stimuli 48 h after treatment (A–D). The surface of area covered by GFAP immunoreactivity was plotted and each scratch was evaluated with an average of four photographs, and each treatment group represented five to six replicates (bar graph). Data are representative of at least three independent experiments ± SEM. One-Way ANOVA followed by Dunnett's post-test: *p<0.05, **p<0.01, ***p<0.001.

Because of the potential role of ERK signaling in cell migration, a scratch assay was then performed to investigate the role of LPS-induced activation in the migratory behaviour of astrocytes in response to S1P. Astrocytes were allowed to migrate for 48 h after treatment with LPS (100 ng/ml), S1P (500 nM) or a combination of both in a scratch assay. Treatments were repeated after 24 h without changing the medium. As shown in Figure 6, all treatments significantly increased migration of astrocytes into the scratches when compared to untreated cells (Fig. 6A). When LPS and S1P were combined, astrocyte migration was not significantly increased compared to migration induced by LPS or S1P alone (Fig. 6D).

**Figure 7:**
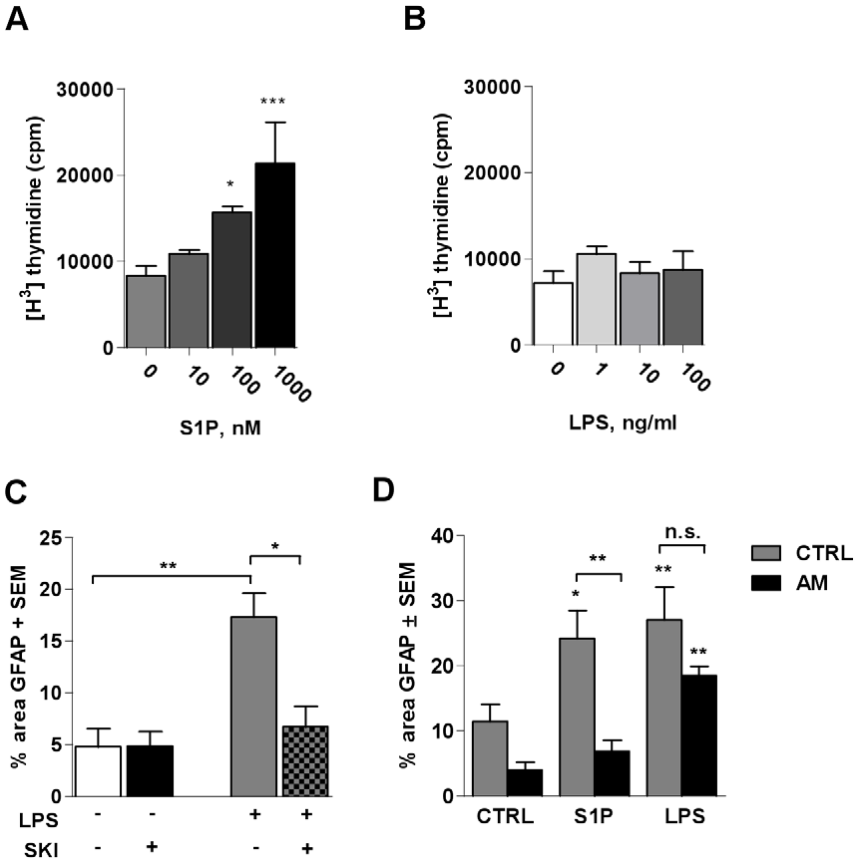
LPS-induced astrocyte migration is SphK1-dependent, but proliferation-independent. Astrocyte were treated with increasing concentrations of S1P (10 nM, 100 nM, 1000 nM) (A) or LPS (1 ng/ml, 10 ng/ml, 100 ng/ml) (B), and cell proliferation was measured using a [^3^H]- thymidine uptake assay. Data are representative of three independent experiments ± SEM. One-Way ANOVA followed by Dunnett's post-test: (A) *p<0.05 vs. control, ***p<0.001 vs. control. Astrocytes were pre-treated or not with SKI (10 µg/ml, 1 h) and then stimulated with LPS (100 ng/ml) (C) or stimulated with S1P (500 nM) and LPS (100 ng/ml), respectively, in the presence or absence of 10 µM antimitotic treatment (D). The graphs show the SKI-mediated inhibition of LPS-induced migration (C) and the influence of antimitiotic treatment on S1P- or LPS-induced migration after 48 h incubation (D). The surface of area covered by GFAP immunoreactivity is plotted. Each scratch was evaluated with an average of four photographs, and each treatment group represented five to six replicates. Data are representative of three independent experiments ± SEM. One-Way ANOVA followed by Dunnett's post-test: (C) *p<0.05, **p<0.01; (D) *p<0.05 **p<0.01 vs. respective control.

Long-term treatment with the S1P_3_ agonist (Compound 20) caused cells to detach, thus the role of the S1P_3_ receptor in astrocyte migration in LPS-stimulated astrocytes could not be assessed directly. We instead investigated whether LPS-induced migration depends directly on SphK1 activity. Cells were pre-incubated with SKI (10 µg/ml) 1 h before performing the scratches, followed by treatment with LPS (100 ng/ml) and they were then allowed to migrate for 48 h. Pre-incubation with SKI significantly inhibited LPS-induced migration of astrocytes (Fig. 7C) whilst not affecting cell survival, as assessed by visual observation and lack of LDH release in the supernatants (data not shown).

S1P was previously reported to be mitotic [33], and ERK signaling is involved in cell proliferation. Therefore we investigated if this was also the case in our cultures by stimulating the cells for 24 hours with increasing concentrations of S1P or LPS in the presence of [^3^H]-thymidine. Figure 7A shows the dose-dependent increase in astrocyte proliferation in response to S1P. In contrast LPS has no effect on astrocyte proliferation (Fig. 7B).

In order to define if the S1P-induced astrocyte migration depends on proliferation, the scratch migration assay was repeated as before, but in presence or absence of an antimitotic agent (Cytosine β-D-arabinofuranoside hydrochloride, 10 µM) (Fig. 7 D). Data show that the astrocyte migration in the control wells was partially dependent on proliferation, as cells cover less area of the scratch in the presence of the antimitotic factor. Similarly, S1P-induced migration was also strongly dependent on astrocyte proliferation and significantly reduced in the presence of the antimitotic factor compared to S1P alone. In contrast, the migration induced by LPS did not depend significantly on proliferation as there was no significant difference between cells treated with LPS, or with LPS and the antimitotic factor.

### LPS and S1P induce CXCL1 release

The release of cytokines and chemokines was shown to be induced by stimulation with S1P in different cell types [6], [34]. In order to better define the link between TLRs and the SphK1/S1P axis, cytokines and chemokines produced by astrocytes in response to stimulation with LPS (10 ng/ml, 100 ng/ml, 1 µg/ml) or S1P (100 nM, 1 µM, 5 µM) were analyzed by a Multiplex ELISA for a total of 23 different factors. Following 24 h stimulation, CXCL1 was shown to be the factor most induced by both stimuli (data not shown). Other factors induced by both stimuli were RANTES and monocyte chemoattractant protein-1 (MCP-1), but to a lesser extent (data not shown).

**Figure 8:**
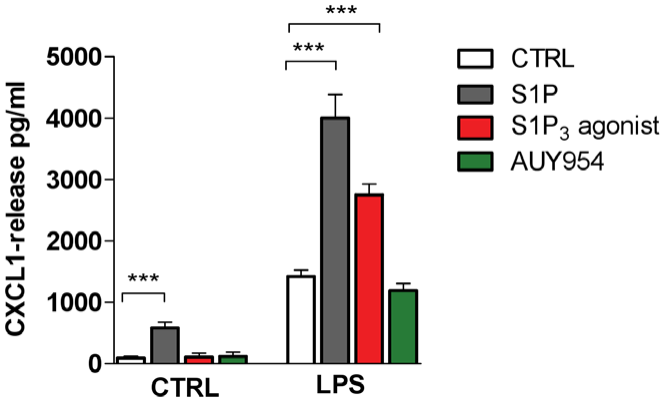
S1P_3_ contributes to the S1P-induced CXCL1 release by primary astrocytes. Astrocytes were pre-treated with or without LPS (100 ng/ml) for 12 h in serum-free medium and then stimulated or not with S1P (1 µM), a S1P_3_ agonist (Compound 20, 10 µM) or a S1P_1_ agonist (AUY954, 10 µM) for 5 h. The graph shows the effect of LPS pre-treatment on the release of CXCL1 by S1P, S1P_3_ or S1P_1_ agonists. Data are a pool of two independent experiments each performed in six replicates ± SEM. One-Way ANOVA followed by Bonferroni's multiple comparison test: **p<0.01, ***p<0.001.

Because CXCL1 has potential neuroprotective effects on oligodendrocytes, we chose to focus the next experiments on the regulation of its release through S1P_1_ or S1P_3_ receptors, following pre-activation with LPS. Astrocytes were treated or not with LPS (100 ng/ml) for 12 h, followed by stimulation with S1P (1 µM), the S1P_3_ agonist Compound 20 (10 µM) or the S1P_1_ agonist AUY954 (10 µM) in fresh medium for another 5-hour period. CXCL1 released into the supernatant was then measured by ELISA. Cells pre-treated with LPS for 12 h followed by medium replacement showed increased CXCL1 release without any further activation, indicating that LPS-induced activation lasts for several hours (Fig. 8). When S1P was given after pre-stimulation with LPS, CXCL1 release was further increased by 3-fold. Furthermore, although no induction of CXCL1 in response to the S1P_3_ agonist was measured in non-activated cells, CXCL1 release was induced by the S1P_3_ agonist in cells pre-treated with LPS. In contrast to the S1P_3_ agonist, AUY954 did not further increase CXCL1 release induced by LPS challenge itself. These data demonstrate that the LPS-induced increase of S1P_3_ receptor leads to increased functionality, as CXCL1 release induced by S1P in activated astrocytes is mediated mainly via the S1P_3_ rather than the S1P_1_ receptor.

## Discussion

We confirmed in this study an increased expression of the S1P_3_ receptor on reactive astrocytes in actively demyelinating and chronic-inactive MS lesions, as described recently [30]. In addition, we showed for the first time that the enzyme SphK1 is strongly upregulated in macrophages, but also on reactive astrocytes in both types of MS lesion. Furthermore, both S1P_3_ and SphK1 expression was shown to be strongly expressed in perivascular inflammatory cell cuffs in MS brain. In contrast, little or no expression of SphK1 and S1P_3_ was seen in normal control brain.

In MS, macrophages infiltrate the CNS parenchyma where they contribute to tissue damage, release of pro-inflammatory mediators, impairment of BBB functions and also mediate damage and phagocytosis of myelin, oligodendrocytes and axons. SphK1 activity was shown to drive macrophages towards a pro-inflammatory phenotype, inducing the release of pro-inflammatory stimuli which can cause further tissue damage [35]. Additionally, SphK1 is upregulated by excitotoxicity and hypoxia in astrocytes and glioma cells, respectively [15], [16]. Both types of injury probably contribute to neuronal damage in MS tissue [36], and increased S1P production in the CNS could well be an additional player in the neurodegenerative cascade by increasing astrogliosis. Our finding of high SphK1 expression by infiltrating macrophages and reactive astrocytes in MS lesions suggests that inhibition of SphK1 activity may be beneficial in MS by dampening macrophage- and astrocyte-mediated inflammation and tissue damage. This is a potential additional benefit of the new MS drug FTY720 as it was recently shown to inhibit SphK1 [37].

In agreement with the study by van Doorn et al. [30] S1P_3_ was not substantially expressed on macrophages in MS lesions, and a recent study showed that in human donor macrophages and microglia S1P_3_ is expressed only at relatively low levels [37]. We therefore chose to focus on the role of the SphK1/S1P_3_ axis in activated astrocytes.

Upregulation of receptor S1P_3_ mRNA in CNS tissues was previously demonstrated in EAE [17], although the identity of the cells overexpressing S1P_3_ was not elucidated due to the lack of specific antibodies against murine S1P_3_ for immunohistochemistry.

The upregulation of S1P_3_ receptors on astrocytes was previously described in a mouse model of the neurodegenerative Sandhoff disease, a prototypical lysosomal storage disorder [38], [39]. It was demonstrated that both S1P_3_ and SphK1 deficient mice had a milder disease course with decreased proliferation of glial cells and less pronounced astrogliosis, clearly indicating a role of the SphK1/S1P_3_ axis in the neurodegenerative process [39]. However, it remains to be shown if this is also the case in EAE. An additional level of complexity in interpreting potential benefit of FTY720 via S1P_3_ activation comes from the fact that the fate of this receptor upon activation is not well understood, as it could either be internalized and recycled or internalized and degraded. The fate of the internalized receptor is important within the context of FTY720, as it may determine whether the drug works as an agonist or a functional antagonist [40].

In line with the increase of SphK1 and S1P_3_ expression on reactive astrocytes in MS lesions, we showed in an *in vitro* system using rat primary astrocyte cultures that SphK1 and S1P_3_ mRNA and protein levels were upregulated when stimulated by LPS. S1P_3_ and SphK1 mRNAs were markedly upregulated five hours after treatment. Interestingly, mRNA levels of S1P_1_ and SphK2 were not modulated in rat astrocytes, thus differing from the study by van Doorn et al. [30] where TNFα increased the mRNA both of S1P_1_ and S1P_3_ in human astrocytes, thereby indicating potential species differences. Alternatively, this difference could come from a distinct effect of TNFα in comparison to LPS, but upregulation of both S1P_1_ and S1P_3_ by LPS was described in human gingival epithelial cells [41]. Astrocyte activation occurs through several different pathways, thus TLR activation by LPS can certainly drive different cellular responses other than those elicited other pro-inflammatory stimuli, such as cytokines. The concomitant regulation of SphK1 and S1P_3_ was demonstrated to play a role in murine cardiac fibrosis [42] and in transdifferentiation of myoblasts into myofibroblasts [43]. Furthermore, knocking down SphK1 by siRNA led to reduced S1P_3_ mRNA expression in MCF-7 Neo cells [44]. As a result, ERK-1/2 phosphorylation via S1P/S1P_3_ was decreased, thereby abrogating establishment of the migratory phenotype. An increased S1P_3_ and SphK1 expression could conversely lead to an enhanced migratory phenotype. All these data clearly indicate that SphK1 and S1P_3_ can be functionally linked, and suggest the involvement of ERK signaling in cell migration.

Various growth factors or pro-inflammatory stimuli [45] including LPS [7] have been shown to activate SphK1, but we show here for the first time that this is also the case in primary astrocytes, as demonstrated by the increased expression of SphK1 protein at the plasma membrane fraction and further verified by direct measurement of its activity.

Several laboratories have described signaling of S1P receptors in astrocytes *in vitro* showing that S1P receptor stimulation by S1P results in phosphorylation of ERK-1/2, a pathway often associated with cell proliferation or migration [44], [46]–[48]. In addition, Osinde et al. [49] showed that the S1P receptor agonist FTY720P also induced phosphorylation of ERK-1/2 in astrocytes, which was assumed to be predominantly mediated via S1P_1_ activation. However, in their study, the S1P-mediated ERK-1/2 phosphorylation was not inhibited by a S1P_1_ specific antagonist, but only by a S1P_1_/S1P_3_ specific antagonist, indicating that S1P-mediated ERK-1/2 phosphorylation occurred not only via S1P_1_ but also via S1P_3_ receptors.

All aforementioned studies were performed under basal conditions, i.e. without activation of astrocytes by pro-inflammatory stimuli. To address the consequences of the upregulation of S1P_3_ receptor by LPS, we used the selective S1P_3_ agonist Compound 20 [32] to assess the magnitude of downstream signaling. We demonstrated an increased S1P_3_-mediated ERK-1/2 phosphorylation in LPS-treated rat astrocytes when compared to non-activated cells. This enhanced phosphorylation was observed only after stimulation of S1P_3_ and not of S1P_1_ (data not shown), indicating that S1P_3_ signaling is preferentially increased in astrocytes under pro-inflammatory conditions *in vitro*. This was further supported by our data showing that LPS did not lead to an increased expression of S1P_1_ receptor mRNA. Phosphorylation of Akt, another well-studied signaling pathway downstream of S1P receptors, is associated with cell survival. The S1P_3_ receptor was shown to mediate cytoprotective effects via Akt upregulation in human endothelial cells in response to vascular endothelial growth factor (VEGF) [50]. We show here the direct activation of Akt via S1P_3_ activation in response to a S1P_3_ agonist, but the S1P_3_-mediated Akt signaling was not further increased in LPS-activated astrocytes, suggesting that out of the two investigated pathways, ERK-1/2 is dominant over the Akt pathway in LPS-activated astrocytes.

ERK-1/2 is an important player in migration or proliferation. The SphK1/S1P axis has also been implicated in migratory behaviour of different cell types, but some studies show that it has a positive effect on migration [21], [44], whereas others show that it is inhibitory [51]. This may depend in part on which of the S1P receptor is involved in signaling, as was suggested by a study which showed that S1P_1_ was driving migration in endothelial cells, whereas S1P_2_ was responsible for inhibition of migration [52].

In order to explore the role of the SphK1/S1P axis in the migration of astrocytes, we performed a scratch assay, as described by Mullershausen et al. [21], and found that both LPS and S1P induced astrocyte migration, but LPS and S1P together did not exhibit any additive effect. Migration in this assay could occur through motility of the cells, but also through cell division. S1P has been described previously as having a mitotic effect on astrocytes through ERK activation [33], further suggesting a contribution of proliferation to our migration assay. A direct proliferation assay assessing ^3^H-thymidine incorporation confirmed that S1P induced astrocyte proliferation, whereas LPS did not. The use of an antimitotic factor in the scratch assay further showed that the LPS-induced migration differs from the migration induced by exogenous S1P, the latter being largely mediated through invasion of the scratch by dividing cells, whereas this was not the case for LPS. Because LPS-induced migration is prevented by inhibition of SphK1, endogenous S1P is likely involved in the migration, but not through proliferation. Long-term treatment with the S1P_3_ agonist caused cells to detach, thus we were not able to directly assess the role of this receptor following LPS treatment.

The hypothesis of Long and colleagues [44] which suggests that increased S1P_3_ and SphK1 expression can lead to enhanced migration could thus not be verified for astrocytes, but a link between both signaling pathways is nevertheless supported by our observation that LPS-induced migration was dependent on SphK1 activity as demonstrated by the use of a SphK1 inhibitor. It is now known that SphK1 can translocate to other compartments than plasma membranes, suggesting that S1P is produced locally in these compartments to act directly on intracellular targets [53], [54], independently of S1P receptors. Interestingly, a recent paper by Berdyshew et al. [55] demonstrates that production of intracellular S1P is essential to induce the motility of lung endothelial cells, even though in their case it seemed to be through release of S1P and signaling from outside the cells. We were not able to measure extracellular levels of S1P in our culture system, thus it has not been possible to assess whether LPS induced the release of S1P through the increased activation of SphK1. However, because exogenous S1P, at a relatively high concentration, was able to induce proliferation, whereas LPS did not affect proliferation at all, we would argue that the S1P produced through the LPS-induced SphK1 activation signals differently than exogenous S1P, and likely increased cell motility rather than proliferation, since inhibition of SphK1 resulted in decreased LPS-induced migration in which proliferation does not play a role.

S1P induces release of growth factors such as FGF-2 or GDNF in astrocytes [56], [57], which could act in a paracrine fashion as neurotrophic factors. On the other hand, S1P has previously been implicated in a number of pro-inflammatory processes. For example, S1P induced an increase in expression levels of TNFα, IL-1β, and iNOS and release of TNFα and NO by activated microglia [6]. It was also shown to increase expression of pro-inflammatory IL-8 and MCP-1 mRNA in human umbilical vein endothelial cells through S1P_1_ and S1P_3_ [34].

In our study we found that MCP-1, RANTES and CXCL1 are secreted by cultured primary rat astrocytes in response to S1P and LPS, with CXCL1 showing the strongest induction. All three factors are known to induce neutrophil and monocyte/macrophage recruitment into lesions, but only CXCL1 has been described as having potential beneficial effects for oligodendrocytes. In LPS-activated astrocytes, S1P-induced CXCL1 release was significantly increased compared to non-activated cells. Furthermore we showed that a S1P_3_ agonist, which did not by itself induce CXCL1 release in non-activated cells, significantly induced release of this chemokine when cells were pre-activated with LPS. This direct induction of the chemokine CXCL1 in response to stimulation of S1P_3_ in LPS-activated astrocytes is coherent with our observation showing a concomitant increase of S1P_3_-mediated signaling in LPS-stimulated astrocytes. Furthermore, the S1P_1_ selective agonist AUY954, which did not increase ERK-1/2 phosphorylation in LPS-stimulated astrocytes, also did not induce CXCL1 release.

CXCL1 is described as a rat pro-inflammatory chemokine with structural and functional homology to human interleukin-8 (IL-8) [58]. It is expressed by inflammatory cells at sites of inflammation and is involved in neutrophil and granulocyte infiltration into the brain [59]. However, CXCL1 has also been shown to induce proliferation of oligodendrocyte precursor cells both *in vitro* and *in vivo* [60], [61], and migration of these cells *in vivo* is arrested in its presence [62]. Furthermore, mice that inducibly overexpress CXCL1 under control of the astrocyte-specific gene GFAP showed neuroprotection and remyelination in EAE [63]. The same group also showed that CXCL1 was produced in MS tissues by reactive astrocytes in close proximity to oligodendrocytes, which express the CXCL1 receptor CXCR2 [64]. CXCL1 release by activated astrocytes could thus act not only as a pro-inflammatory chemokine, but could also exert beneficial effects on remyelination in MS. Our data emphasize the role of S1P_3_ rather than S1P_1_ in increased CXCL1 release by activated astrocytes, which probably involves ERK-1/2 signaling, as suggested by previous studies that had shown the role of ERK-1/2 in TLR-4 mediated release of IL-8 and MCP-1 in adipocytes and in lung epithelial cells [65], [66].

In summary, our study demonstrates that SphK1 and S1P_3_ expression, signaling and biological responses are increased in LPS-activated astrocytes. Reactive astrocytes in MS lesions also showed increased expression of SphK1 and S1P_3_, suggesting that this signaling axis may play a role in mediating and amplifying inflammatory responses in various CNS disorders in an autocrine/paracrine fashion. Conversely, it may also lead to beneficial effects on remyelination through release of CXCL1. How S1P_3_ signaling in astrocytes induced by FTY720 could influence the disease course in MS patients remains to be elucidated but inhibition of SphK1/S1P_3_ signaling on astrocytes via inhibition of SphK1 activity could result in reduced neuroinflammation.

## Materials and Methods

### S1P_3_ expression in MS and control CNS tissues

Samples from cases clinically diagnosed as MS and confirmed by neuropathological examination were provided by the NeuroResource tissue bank (UCL Institute of Neurology, London, U.K.). Informed written consent from donors and/or their next of kin was obtained for the donation of MS and normal control CNS tissues to the NeuroResource tissue bank for research studies. The NeuroResource tissue bank documentation for tissue donor information and the consent forms for tissue donation were approved by the Central London Research Ethics Committee 1, London, U.K.. Permission was given by the NeuroResource MTA Review Committee based at the UCL Institute of Neurology for samples from the tissue bank to be used in this specific study. An application for ethical permission to carry out this study on human CNS tissues was approved by L'Association des Médecins du canton de Genève et Société Médicale: Commission d'éthique pour la Recherche Clinique en Ambulatoire, a Research Ethics Committee based in Switzerland.

Cryostat sections (10 µm) were cut from 8 blocks of snap-frozen MS lesions with adjacent tissue from the cerebrum of 7 MS patients (average age 51 y (range 29–69 y); clinical disease duration 21 y (range 8–34 y); interval between death and tissue snap-freezing 15 h (range 8–24 h)). Sections were also cut from 5 blocks dissected from the cerebrum of 4 age-matched control cases without CNS disease (average age 57 y (range 47–68 y); interval between death and snap-freezing 19 h (range 11–23 h)). The tissue blocks were dissected from periventricular or subcortical white matter in frontal, parietal or occipital lobes and cerebellum. Anti-myelin basic protein (MBP) (1∶100; Cat. No. MAB382, Chemicon, Temicula, CA) immunostaining of all MS lesions was performed to assess demyelination (not shown). Oil red O and haematoxylin histological screening (not shown) further demonstrated that 5 lesions were actively demyelinating, 1 was chronic-active and 2 were chronic-inactive.

For immunostaining sections were fixed (ice-cold acetone, 10 min), rinsed in phosphate-buffered saline (PBS) and blocked in 10% normal goat serum and 1% bovine serum albumin (BSA; low endotoxin, fatty acid free; Sigma, MO, USA) and incubated (1 h, RT) with either a polyclonal anti-S1P_3_ antibody (1∶100; Cat. No. AB9289, Millipore, Temecula, CA) [30] or a polyclonal anti-SphK1 antibody (1∶50; Cat. No. AP7237c, Abgent Europe; Oxfordshire, UK) [44]. Sections were then incubated with biotinylated anti-rabbit antibody (1∶500; 1 h, RT) and then for 1 h with A+B from a Vectastain ABC Kit (all from Vector Laboratories Inc., California). Immunoreactivity was visualized by NovaRed peroxidase substrate (Vector Laboratories), followed by dehydration and cover-slipping with Eukitt™ (Kindler GmbH & Co, Freiburg, Germany). To identify cell types expressing S1P_3_ and SphK1 double-immunostainings were performed by incubating sections with mouse anti-GFAP (1∶200; Chemicon, Temicula, CA) or mouse anti-CD68 (1∶250; Dako, Glostrup, Denmark) with either anti-S1P_3_ or anti-SphK1 (1 h, RT) followed by goat anti-rabbit coupled to Alexa 488 (1∶200) for anti-S1P_3_ or SphK1 and goat anti-mouse coupled to Alexa 555 (1∶200; Invitrogen, Basel, Switzerland) for anti-GFAP or anti-CD68. Sections were mounted in FluorSave™ (Calbiochem, San Diego, CA) and examined on a Leica SP2 confocal microscope.

### Animal studies

All the animal work was carried out after being approved by the internal Merck Serono Ethical Committee and the cantonal veterinarian office (license numbers 1040/3123/0-R and 1040/3123/0-2R), as well as by the federal veterinarian office, according to the Swiss Law and on Animal Protection (2008, 2009) and the Swiss ordinance on Animal Experimentation (2010).

### Rat astrocyte cultures

Glial cells were isolated from newborn OFA (Oncins France Strain A) rat cortices essentially as described previously [67]. Briefly, brains were collected in dissection medium (HBSS, penicillin/streptomycin, HEPES 10 mM, sodium bicarbonate 0.75% (all from GIBCO/Invtirogen). Cortices were cut into small pieces and dissociated in 10 ml dissection medium containing 0.01% trypsin and 10 µg/ml DNAse I (Sigma; 10 min, 37°C). Following centrifugation (100× *g*, 5 min), the pellet was gently dissociated and filtered through a 70 µm mesh, centrifuged and resuspended in culture medium (Dulbecco's modified Eagle's medium (DMEM, Invitrogen), 10% fetal bovine serum, penicillin/streptomycin) and grown on poly-D-Lysine T75 flasks in a 10% CO_2_ 37°C incubator for 10 days with a medium change every 3–4 days. At confluence cells were submitted to two consecutive shaking steps to remove microglia and oligodendrocyte progenitor cells. The remaining astrocytes were washed with PBS and detached with PBS-EDTA incubation followed by trypsin-EDTA treatment, and plated according to experimental requirements.

### Determination of mRNA levels by real-time PCR (qPCR)

For RNA isolation 3×10^5^ cells were grown in culture medium for 2 days in six-well plates and then treated with 100 ng/ml LPS (from *Escherichia coli* 0111:B4; Sigma) for 5 h. Total RNA was isolated using a RNeasy microkit (Qiagen, Valencia, CA) and reverse-transcribed using a iScript™ cDNA Synthesis Kit (Bio-Rad, Hercules, CA). Quantitative real time PCR was performed using a SYBR green PCR kit (Roche, Basel, Switzerland) and PCR products were detected using an ABI PRISM 7900 sequence detection system (Applied Biosystems, Foster City, CA). Primers used to amplify S1P_1_, S1P_3_, SphK1 and SphK2 were from Qiagen (S1P_1_: QT00441007; S1P_3_: QT00440909; SphK1: QT00182035; SphK2: QT01783145) and expression of these transcripts was quantified against the housekeeping gene glyceraldehyde 3-phosphate dehydrogenase (GAPDH), which was amplified using the primers 5′-GGAGACAACTGGTCCTCCAGTG-3′ and 5′-ACCTGCCAAGTATGATGACATCA-3′. GAPDH expression was used as a housekeeping gene as it was not modulated by the treatment with LPS (data not shown). Expression levels of target genes were analyzed using the SDS 2.2.2 software system (Applied Biosystems, Foster City, CA).

### Western blot analyses

To evaluate phosphorylated ERK-1/2 and phosphorylated Akt, 3×10^5^ cells in 6-well poly-D-lysine plates were grown for two days in culture medium. They were then pretreated for 12 h with LPS (100 ng/ml) in serum-free medium, followed by 20 min stimulation with the S1P_1_ selective agonist AUY954 [68] or 10 µM of the S1P_3_ selective agonist Compound 20 described by Schurer et al. (2008) [32]. Compound 20 is a dicyclohexylamide and a nanomolar selective S1P_3_ agonist with an EC50 value of 0.35 µM, and is inactive against S1P_1_, S1P_2_, S1P_4_ and S1P_5_. Both compounds were synthesized as previously described [32], [68]. Subsequently cells were lysed in 50 mM Tris/HCl buffer pH 8.0 containing 150 mM NaCl, 0.02% sodium azide, 0.1% SDS, 1% IGEPAL Ca-630, 0.5% sodium deoxycholate, 40 mM β-glycerophosphate, 1% NaF, 0.1% sodium orthovanadate and protease inhibitor mixture tablet (Roche). After a brief sonication protein concentration was determined using a BCA kit (Pierce, Rockford, IL) and 20 µg of protein in sample buffer containing DTT (Sigma) was loaded on a SDS polyacrylamide gel (Invitrogen, NuPAGE Novex Bis-Tris gels, 4–12%) for electrophoresis, and transferred to a nitrocellulose membrane. Membranes were blocked using 5% BSA/0.05% Tween-20®/PBS for incubation with polyclonal antibodies against phosphorylated ERK-1/2 (1∶1000) and phosphorylated Akt (1∶500; Cell Signaling Technology, Boston, MA), or 5% non-fat dry milk/0.05% Tween-20®/PBS for a monoclonal antibody against β-actin (1∶5000; Millipore), for 1 h and then probed with these antibodies overnight at 4°C. Immunoreactive bands were detected with appropriate horseradish peroxidase-conjugated secondary antibodies and an ECL detection kit (GE Healthcare, Little Chalfont, U.K.).

An anti-SphK1 polyclonal antibody (Cell Signaling Technology, Cat. No.:3297s; 1∶700) [69] and a polyclonal anti-S1P_3_ antibody (1∶200; Cat. No.: 10006373, Cayman Chemicals, Tallinn, Estonia) [70] were used to immunodetect SphK1 and S1P_3_ in membrane fractions. Equal loading of protein was controlled by expression of β-actin after membrane stripping.

### Membrane fractionation

Cells were plated on 10 cm poly-D-lysine coated Petri dishes in culture medium (2×10^6^ cells/dish) until they reached 90% confluence. Serum was replaced 12 h before stimulation with LPS (100 ng/ml) for the indicated time points. The reaction was terminated by washing the cells twice in ice-cold PBS. Then the cells were scraped into ice-cold PBS, centrifuged (5 min, 1000× *g*), the supernatant was removed and cells were triturated with a 25G needle in TBS (50 mM Tris-HCl, pH 7.4, 150 mM NaCl) and the lysate was centrifuged (30 min, 184,000× *g*). The supernatant was removed and membrane proteins were solubilised by trituration with a 25G needle in TBS/1% Triton X-100 containing EDTA-free protease inhibitor (Roche). The lysate was incubated on ice for 15 min with repeated vortexing. Insoluble debris was removed by centrifugation (10 min, 20,000× *g*, 4°C) and supernatant protein concentrations were determined by BCA (Pierce). Samples (20 µg) containing DTT and running buffer were heated (60°C, 3 min) and loaded on a 10% SDS-page gel for electrophoresis and Western blot analysis.

### SphK1 activity assay by thin layer chromatography

SphK1 activity was measured according to Olivera [71] with a few modifications. Briefly, cells were plated on 10 cm poly-D-lysine coated Petri dishes in culture medium (2×10^6^ cells/dish) until they reached 90% confluence. Serum was replaced 12 h before stimulation and the cells than pre-incubated or not for 2 hours with 10 µg/ml of the SphK inhibitor (SKI; (2-(*p*-Hydroxyanilino)-4-(*p*-chlorophenyl)) thiazole HCl (Merck Bioscience, Darmstadt, Germany), followed by stimulation with 500 ng/ml LPS for 45 min. The reaction was then stopped on ice and the cells were scraped from the dishes using 20 mM Tris-HCl, buffer pH 7.4, containing 10% glycerol, 1 mM 2-mercapoethanol, 1 mM EDTA, 1 mM sodium orthovanadate, 40 mM β-glycerophosphate, 0.5 mM 4-deoxypyridoxine, 15 mM NaF, 0.1% Triton X-100 and EDTA-free protease inhibitor mixture (Roche). After brief sonication and determination of protein concentration, 100 µg of protein was incubated in 200 µl final volume of reaction mixture containing 50 µM sphingosine (Avanti Polar Lipids, Alabaster, AL) in 5% Triton X-100, [γ-^32^P]ATP (Perkin-Elmer Life and Analytical Sciences; 10 µCi, 20 mM in 200 mM MgCl_2_) and SphK1 activity assay buffer for 37°C. The reaction was terminated by addition of 20 µl 1 M HCl followed by 400 µl of chloroform∶methanol∶HCl (100∶200∶1, v/v/v) and allowed to stand at room temperature for 10 min, then 125 µl chloroform and 125 µl 2 N KCl were added. Samples were centrifuged (400× *g*, 5–10 min) and the organic phase was dried under a nitrogen stream. The pellet was dissolved in 50 µl of chloroform∶methanol∶HCl (100∶100∶1, v/v/v) and spotted onto a silicon TLC plate which was placed in a TLC developing tank (1-butanol∶methanol∶acetic acid∶water (80∶20∶10∶20, v/v)). S1P radioactive spots were exposed to X-ray film, developed with a Phosphoimager™ and analyzed using Quantity One® software.

### Proliferation Assay

To study the proliferation of astrocytes in response to S1P or LPS, 5000 cells/well were plated in 96-well poly-D-lysine plates over night in culture medium. At this density, cells are not confluent. The next day, they were washed in serum-free medium and then incubated with increasing concentrations of S1P (10, 100, 1000 nM) or LPS (1, 10, 100 ng/ml) in fresh serum-free medium for 24 h. During the last 18 h [^3^H]-thymidine (1 µCi/ml) was added to the wells. The cells were then harvested using a Filtermate Harvester (Packard) and [^3^H]-thymidine uptake was counted using a Microbeta Trilux counter (Perkin Elmer).

### Scratch migration assay

Astrocytes were plated onto 24-well poly-D-lysine plates (3×10^5^cells/well) and incubated overnight in 10% CO_2_ at 37°C. The following day one scratch per well was made using a 1 ml pipette tip, and cells were washed twice in PBS to remove debris. Cells were treated with the different stimuli in DMEM 1.5% horse serum and allowed to migrate into the scratch for 48 h. Treatments were repeated 24 h later without changing the medium. For inhibition studies, cells were pre-incubated with 10 µg/ml SKI (Merck Bioscience, Darmstadt, Germany) for 1 h before performing scratches. After the scratch, the cells were then treated with a single dose of LPS and allowed to migrate for 48 h. To investigate the influence of proliferation on astrocyte migration the antimitotic agent Cytosine β-D-arabinofuranoside hydrochloride (10 µM; Cat. No. C-6645; Sigma) was applied together with LPS (100 ng/ml) or S1P (500 nM). After 48 h, the cells were fixed with 4% paraformaldehyde, washed and permeabilized with 20% methanol and then with 0.5% Tween 20®. Following washing they were incubated with rabbit anti-GFAP antibody (1∶200; Cat. No. Z0334, DAKO) for 1 day at 4°C and then incubated with a goat anti-rabbit Alexa 555 1∶250 (Invitrogen) and Hoechst 1∶10,000 (Sigma) in PBS for 1 h. Cells were washed and four photographs per scratch were taken with a fluorescence microscope in a blinded fashion. The percentage of cells which migrated into a scratch was determined by analyzing the percentage of area positive for GFAP using ImageJ software.

### CXCL1 protein determination

CXCL1 protein, also called GRO (Growth regulated oncogene) or CINC-1 (Cytokine induced neutrophil chemoattractant-1), secreted by astrocytes was measured using a rat CXCL1 ELISA kit (GRO/CINC-1C ELISA Kit, R&D Systems) following manufacturer's instructions. Astrocytes were plated onto 96-well poly-D-lysine plates (2×10^4^ cells/well) for one day then the culture medium was replaced by serum-free medium containing LPS (100 ng/ml) for 12 h. The next day cells were treated with a S1P_3_ selective agonist (Compound 20, 10 µM), S1P (1 µM; Avanti Polar Lipids) or the S1P_1_ selective agonist AUY954 (10 µM) for 5 h, and culture medium was collected and analyzed by ELISA.

### Data plotting and statistical analyses

All data were plotted using GraphPad Prism version 5.02 (GraphPad Software, San Diego California USA). One-way ANOVA was applied to evaluate statistical significances. Post-tests and significances are described in the Figure Legends.
